# Proceedings of the American Dental Association

**Published:** 1875-12

**Authors:** 


### ARTICLE V.

### *The American Dental Association—Fifteenth Annual Session.*

THIRD DAY—*Morning Session.*

The association was called to order at the usual hour, the president in the chair.

A motion was offered providing for the appointment of a committee to confer with a similar committee of the American Dental Convention in regard to a joint meeting next year at Philadelphia. Laid on the table.

On motion of Dr. J. H. Smith, a committee was appointed to draft appropriate resolutions in regard to the death of Dr. Hill, of Norwalk. Carried, and Drs. Smith, Abbott, and Harlan appointed

The subject of " Dental Chemistry " was then taken up. This subject had been opened on the previous evening, before adjournment, by the reading of a report from the committee by Dr. H. A. Smith.

We give the following synopsis:

The report first alluded to a subject which had been investigated during the past year by Dr. S. B. Palmer, a member of the committee: viz.: " Chemical and Galvanic Action upon the Teeth and the Materials used in filling." Dr. Palmer assumed that many cases of failure after careful filling are due to an electro-chemical decomposition of the tooth-bone, induced by a current set up between the tooth-bone and the filling material He claims that caries is a result of chemical action, and that fillings merely prevent it by shutting out the fluids from the cavity. He believes that in some specific cases other materials than gold will be more effective in arresting this action. The circumstances which seem to give support to this view are, that the latent electricity contained in all matter is liable ot be developed, under favorable conditions (as in a fluid of a certain character,) into a current; and this effort to restore the equilibrium will result in a loss of both form and texture in one of the elements. Changing one of the elements in a battery for one of a higher order will cause a reversal of polarity ; a change of the solution from acid to alkali also changes the polarity. Tooth-bone and filling materials stand in the following electrical order when tested by the galvanometer, with saliva for the fluid ; gold, mercury, oxychloride, dentine, tin, amalgam. In weak acid the order is reversed in some of the elements, and gold and dentine stand at the extremes of the list, the latter becoming the positive element or element acted upon. Between the materials at the extremes of the list the greatest action will take place, and between those nearest each other the action will be least,—so slight, in fact, that a change of fluid, or even a change of temperature in the same fluid, will cause a reversal of the action. In an abnormal or porous condition of the tooth-bone, gold by reason of its negative properties, supplies a decomposing current, while the lower grades of filling materals being more nearly upon the same plane of equilibrium with dentine, less action is produced and less breaking down of the tooth-structure. Tin not only arrests decay mechanically, but in frail, chalky structures acts as an antacid element, arresting the current. Gold is best in a well-organized tooth. Oxychloride, though nearly neutral as regards tooth-bone, is subject to chemical action itself, and hence not permanent. Amalgam stands higher in the scale, and, like gold, is liable to excite a chemical action. If there is not a perfect combination the mercury will be acted upon by the current induced by the finer element, and the effect of such action is visible in the fine pits which we see upon the surface and edges of the plug. Any attempt to make the amalgam finer is, therefore, a step in the wrong direction.

The report then touched upon the amalgam question as presented in the late papers of Drs. Hitchcock and Bogue. It was stated that while the physical properties of amalgam are of the greatest importance, yet the chemistry of amalgam must take precedence; for though we might obtain an article free from expansion or discoloration, yet if these compounds affect the system injuriously, the question of their use would be at once settled.

The experiments of Dr. Bogue where then alluded to, by which it was shown that three months of chemical test failed to materially diminish the weight of the test-plugs; and also that the fluids which contained the teeth and pellets, upon being tested for mercury, were found free from it. From these results Dr. Bogue had concluded that almost any amalgam, if used intelligently, could be employed without danger to the general health. The different conclusions reached by Drs. Palmer and Bogue indicate irreconcilable differences of opinion; but when opinions are based upon demonstrations and are free from prejudice, progress is being made, and ere long the question may be settled.

Prof. Cassidy, a member of the committee, also contributed some observations upon the subject of bleaching teeth. He reviewed the chemical agents which cause discoloration in decayed teeth; the precipitated sulphides, being mainly insoluble, are considered as a principal source of discoloration. The writer considered that teeth can in general be restored to a normal color; but caution is necessary in the use of bleaching agents, else they may effect the tooth-structure injuriously. The most successful treatment is found to be the application of chlorine gas, which is generated in a small retort or flask, by the action of hydrochloric acid upon the black oxide of manganese. Upon applying a gentle heat the chlorine will be developed, and may be conveyed to a receiver or bottle, from which it may be applied to the tooth by means of a straight glass tube some inches in length, one end of which is inserted in the cork of the bottle and the other is drawn to a fine point. Upon inverting the receiver the chlorine will pass downward into the tooth, which must be in a moist condition, as the gas will not bleach otherwise. It should be followed by the immediate use of an alkali, to neutralize any acid, and the mouth and nostrils should be well protected by the dam, to prevent the irritating effects of the gas when inhaled.

The subject being open for discussion—

Dr. Palmer, of Syracuse, whose investigations had been referred to in the report, made some remarks with a view of further explaining his theory in regard to dental caries. There are, he said, two distinct divisions of teeth calling for our operations; and they call, in his opinion, for different methods of treatment. First, those which, though decayed, may be considered as in a normal condition as regards their structure. For such teeth the operation of filling is purely mechanical; we plug the cavity and exclude the moisture. The dentine is in a normal condition, and the gold, though of opposite electrical condition, does no harm in contact with the bone. But there is another class of teeth which demands antiseptic treatment. For instance, the sixth-year molars, when soft and chalky; in these teeth gold is not only not the best, but it is positively injurious.

The speaker then went into a minute exposition of the principles of galvanism, as applied to the case in point. In the galvanic cell there are two elements, the higher and the lower. When gold and tooth-bone are brought in contact these elements are present, and the relations of the battery are sustained. The idea had been entertained that only metals would form such relations, or, in other words, act as conductors; but experiments had proved that tooth-bone was such a conductor. In firm teeth it is a non-conductor, but in soft, porous teeth it becomes a conductor, and the fillings become loosened. We can measure this action with the galvanometer. Tin possesses antiseptic properties which gold does not possess. Fletcher says it is the plasticity of the tin, but it is more than that. The tin stands in almost the same electrical relation as dentine, and, when submitted to the galvanic action spoken of, it is consumed and not the dentine.

The two substances, tin and dentine, however, being so nearly of the same condition, there is very little galvanic action. Amalgam, to his surprise he had found to stand nearer to gold than tin in this respect, when he supposed it would fall below. Any attempt to make it finer was a step in the wrong direction. He did not recommend amalgam ; the cases are few in which it should be used, but when used it should be in accordance with chemical laws. If tin would stand abrasion it would be better in every case. When amalgam is used there cannot be a perfect union of the metals till after crystallization, and the mercury will remain around the edges, and will be dissolved out, and the edges will be ragged and the mass porous. The mass should correspond in electrical condition with the dentine. In some amalgams this seems to be almost attained.

He had had a case in which a partial gold plate had salvated a patient on account of the different electrical condition of the patient's mouth. He had boiled the plate in strong soda, to produce a different surface, and in a little time the system adjusted itself. This case was one of a thousand, as gold hardly ever affected a patient in that manner. It should be understood that the galvanic current does not destroy, but is only the evidence that chemical action is going on, the result of which must be destruction.

Dr. Taft inquired why it was that when a gold and amalgam filling are in close contact the dentine is apt to be destroyed around the gold.

Dr. Palmer said that it was because there was an alkaline current about the amalgam, but around the gold there was acidity. He could not say whether the chemical action produced the current or the current the chemical action. It did not matter, however, which preceded, for one follows the other on the wings of lightning.

Dr. Taft spoke of cases in which there was persistent pain when amalgam fillings were inserted, which continued till their removal, and asked why it was.

Dr. Palmer said that it was in consequence of a difference in electrical condition ; amalgam was not proper in those cases, but gold was.

Dr. Chandler asked Dr. Palmer's opinion as to the plan recommended by Mr. Fletcher to overcome chemical action, viz: to varnish the interior of the cavity with copal, to act as a non-conductor.

Dr. Palmer said that any varnish that would act as a non-conductor would answer the purpose; it fills the pores of undeveloped dentine.

Dr. Osmond. Decomposition around fillings occurs in an acid condition of the buccal fluids. The acids have an affinity for the phosphates of lime. The silver of the old-fashioned amalgams oxideied and combined with the tooth as does nitrate of silver. Amalgams containing a large proportion of tin do not discolor, and the shrinkage is less. He rarely uses amalgam, and is not defending it. He has seen many a good gold filling rendered useless by the proximity of amalgam. As Dr. Watt once said, "A thunder-mill has been erected in the patient's mouth!" Where mouths are very acid there is the most evil. In such cases he varnished the cavity with silicate of soda and touched it with sulphuric acid and water, washing out afterwards, which left a film of silicate, upon which he inserted the filling, and did so without pain. The cavity was protected, and there was no sensitiveness. He had found gratifying results from such treatment.

Dr. Taft said that he had been gratified at the remarks of Dr. Palmer; he does not go so far as he would like, nor so far as he will if he keeps on in the present course. We ought not to be satisfied with the investigations of Dr. Palmer and one or two others, but each ought to investigate for himself. Amalgam has not answered, and it is doubtful whether it will ever be so known as to be used with reasonable expectation of saving teeth. But if it or other materials are to be used, they should be as thoroughly understood as possible. It has been used in the dark by the great mass. The dental depot men had told him that since the papers of Drs. Hitchcock and Bogue on this subject they are selling largely-increased quantities of amalgam. Now, if these papers prove anything, it is that amalgam is unfit to fill teeth with. The directions in which Dr. Palmer had been investigating had not been touched upon at all in these experiments. These are the great questions in regard to it,—not whether it will contract or discolor, but its effect on the system, and the points which Dr. Palmer has been making.

There is here a large and profitable field for investigation. Bleaching teeth is an important subject, and but little understood. We leave out of view the agent producing the discoloration ; it is produced by various things. There is some change of color inevitable when the pulp is devitalized especially in young teeth ; there is a change in the soft solid substances of the tooth. The larger amount of this material the more difficult will it be to restore the color. In some cases excavation will be sufficient. What should be used will depend on what the coloring material is. Almost any agent thus far used will both fail and succeed. Chlorine is the great bleacher, and it will accomplish it so far as it can reach, but no farther. If the tooth is blackened through its substance, nothing will ever remove it. Mistakes are sometimes made in endeavoring to do this. In one case within the speaker's knowledge, after a long treatment with chloride of lime, the entire crown of the tooth came off.

Dr. Rehwinkel said that he was gratified at the remarks of Dr. Palmer, and thanked him for his experiments. This theory explained a great many things for which he had been able to find no satisfactory solution. Looking over his work, and knowing that he never filled without the greatest pains, he finds wear and tear ; the filling is faultless, but the tooth has left the filling ; the closest joint had been undermined. Fillings sometimes remained good for some years, and then there seemed to be set up a morbid chemical action which caused destruction. He believes the oxide of tin possesses a peculiar medicinal action upon tooth-bone, which enables it to resist decay. In children's teeth it is better than gold. His suspicions that the destruction he had observed depended upon causes over which we have no control had been confirmed by Dr. Palmer's remarks. The amalgam question should be handled carefully and with kid gloves. Dr. Bogue and others had shown us that amalgam must stand upon its own merits and not upon the maker's name. He hoped amalgam would never be raised to the dignity of discussion. To some it might be furnished free of charge in unlimited quantities, while it would not be safe to recommend it to others. If we ever undertake to discuss amalgam it will be a curse to the operative department, as rubber has been to the artificial. Still, he would sever his connection with a body which would forbid his using it; when he does so he wants to know why he does it. He looks with dread at the tendency which the question has taken, and sees a cloud rising in the horizon.

Dr. Hunter (Michigan) said that he is practicing in a small town of fifteen hundred inhabitants, where the people had been taught by traveling dentists that amalgam was as good as gold. They would work for half price, and tell people that it was only for looks that gold was used in front teeth. He had himself become disgusted with amalgam, and had put in but one filling of it since 1874; but some of his patients had left his office never to return because he would not use it. They could do work so much quicker too than he could, even with all the facilities for working rapidly, and they could do it without pain. He thought these difficulties which he encountered in a small place were well worthy the attention of the association.

Dr. Daboll spoke of a patient whom he had seen who had teeth filled in Berlin. The appearance of the fillings attracted his attention, and on inquiry and investigation he had discovered that they were a combination of gold and tin. Since that time he had himself used this combination and the result, as observed after two years, has been highly favorable.

The subject was then passed.

There being no report on the subject of "Dental Therapeutics," that of "Operative Dentistry" was taken up and

Dr. Stockton read the report of the committee, written by Dr. McCall and himself.

The following is the substance of the report:

Dr. McCall's report was confined entirely to the department appliances. The burring-engine was spoken of somewhat at length, as standing at the head of the list. The Elliot suspension and S. S. White engines were considered as the leading styles, and the merits of each were spoken of. Ths attachments for injecting air and water were especially mentioned, together with the equipment of burs, disks, polishers, burnishers, etc., and the fear was expressed that many purchasers of engines did not avail themselves of these advantages, but were trying to get along with a scanty outfit.

Motive-powers were then considered; electricity, steam, springs, and water-power being named; the last being considered as having advantages over all the others for those having water in their offices

The various mallets were then spoken of. The ele.tric has its warm friends and staunch opposers. The S. S. White plugger for the engine was named as undoubtedly the best in its line, and driven by the Backus motor, had been used by the writer for a year, and had proved a perfect success. The work has been easier for both operator and patients, and the latter express a decided preference for its use.

Minor appliances, such as Bancroft's and Hickman's rubber dam clamps, Jarvis's separators, Bogue's tape-forceps, and Magill's buckle, were briefly mentioned, and the report concluded with the caution that a few well-seclected instruments would do better service than a multiplicity, which would load down the operating-stand and confuse the operator.

Dr. Stockton's report was upon the same branch of the subject, and was mainly devoted to the electro-magnetic mallet and the Backus water motor, both of which were spoken of in the highest terms. In regard to the former, it was stated that it had received the indorsement of Professors Truman, McQuillen, and Arthur, and Drs. Jack, Webb, and others, as second to no appliance save the rubber dam. One-half to two-thirds the time is saved by it; the most delicate walls are preserved, and every pulsation is under the will of the operator, who is greatly relieved from nervous and muscular exertion.

Three different forms of automatic pluggers—the Buckingham, Gaylor's pneumatic, and Swartley's—were very briefly noticed, and the report concluded with a somewhat enthusiastic mention of the Backus motor, as with its various uses best calculated of all the numerous appliances to preserve the health and prolong the life of the operator; in fact, with that and the rubber dam, and electric mallet, dentistry is made easy, and we would grow young again in its practice, and "return from our vacations with overflowing delights to its welcome embrace."

The subject being open for discussion,—

Dr. Taft said that the report was a good one, but come caution must be exercised lest these new things run away with us. It seems as if all there was in operative dentistry in these days was to have something to bore with. This ought not to be. We should not attempt to do everything with the dental engine. Some do, and bore and grind more than they ought, and with far less precision than could be done by hand. This is more especially so with young men; they fail in delicacy and precision, and work as though the teeth were devoid of vitality. They need a caution, and a special caution. He would not have it understood that he does not like these appliances. While we improve the machines we should also improve our knowledge of the things on which efficient operations on teeth are based. We must know how to do the work, and the condition of the paris, and to control and manage that conditon. We must not run wild on instruments and appliances, but value all things as they deserve.

Dr. Atkinson. Almost all teeth requiring filling have currents of circulation, which must be taken into account; and because they have not been, the pulps have died and the teeth must be bleached. Some poor conductor ought to be substituted for the lost dentine so far as possible. If we could use simply a stratum of gold plate at the periphery of the cavity, which would exclude all the air, it would be a perfect filling. Hill's stopping or oxychloride should be used: but if the latter, it should not be brought into contact with the pulps at all. He mixes the oxide of zinc with salicylic acid, and fills over it with the oxychloride.

Dr. Abbott. Young men should not use the engine at all till they have learned to do the work well without it. They should study the anatomy of the teeth and the distance they can cut. Thousands of teeth have been filled upon a live pulp, and it has died and must be bleached. He does not believe that amalgam enters into the substance of the tooth at all; it is the semi-fluid substances in the tooth which discolor. He had never seen the water motor; the only thing seems to him to be the electric machine sold by S. S. White for running the engine. Had never used a solution of salicylic acid for capping pulps. Pulps may be preserved when oxychloride is in contact with them. Thermal changes are likely to cause the death of the pulp, to prevent which Hill's stopping is most successfully used; never fills without it if there is room to insert it. In root-canals takes a solution of gutta-percha in chloroform and carries it into the canals with a highly-polished instrument, places a piece of Hill's stopping over it, and forces it into the canals. Many canals it is not safe to enlarge; objects to the attempt to enlarge canals in general. Many teeth are ruined by drilling through.

Dr. McKellops said that it was not necessary to enlarge the canals; they could be filled with a solution of gutta-percha without it. He exhibited specimens showing how perfectly the solution would enter the canals. This was said to have been done twenty years ago, but he had never heard or read of it till it was brought forward by Dr. Bowman, of St. Louis. By wrapping a few fibers of cotton on a broach a solution could be carried into the canals, and by placing a piece of softened gutta-percha over it and applying pressure with a larger-pointed instrument the soft material would be forced very perfectly into the canals.

Dr. Rehwinkel. Is it possible that a tissue so delicate as a pulp will tolerate an agent so strong as chloride of zinc, an agent which is used to destroy tissue? How is this to be reconciled? How is it possible that it preserves here while it destroys elsewhere? Dr. Atkinson is more responsible than any other living man for any mischief that has as yet arisen from this treatment, and more harm has been done with it than with any other remedy that we have. Chloride of zinc is a sneaking assassin; it is not entitled to the respect due to the bold attack. If it does not kill, it is because the constitution is so strong as to successfully resist it. It mummifies and keeps things quiet, and appears to be a brilliant success, but the pulp is most likely dead. The reports we hear are of only a few weeks' standing. We do not open and examine, and run the risk of injury. If it does not give trouble, we are justified in thinking it is alive. The condition of the pulp in exposure ought to govern us. We should not always douce with carbolic acid, not at all when freshly exposed. What is the use in cauterizing a wound which we wish to heal by first intention? There is no better substance for capping than gutta-percha in chloroform; if oxychloride is used, would cover the pulp with paper, cork, or something of the kind, and then would risk it. If the pulp is suppurating there is difficulty. These specimens of Dr. McKellops are the best thing he has seen. It has become necessary to defend the use of anything but gold in roots, but gold is not under all circumstances the best either for roots or teeth. When we cannot get out all the pulp, oxychloride may be used to mummify the rest.

Dr. Atkinson said he had been held responsible for the use of oxychloride of zinc, but he wished to announce that he is not responsible for men understanding through their elbows. It is a misunderstanding of plainly-stated truths.

In the DENTAL COSMOS for March, 1868, *Atkinson's* method would be found. It is the same now, except that he uses salicylic acid, the active principle of creasote. He makes a pool of it over the exposure, makes a cream of oxychloride and drops it over it; this drives off the creasote, which is then absorbed, and a filling of stiffer oxychloride is put over it; cover either with gold or apellicle of collodion. He is no assassin, except to error.

Dr. Rehwinkel said he had simply stated a fact; we know that Dr. Atkinson indorses this substance in immediate contact with the pulp.

Dr. Atkinson. I don't; what is immediate contact?

Dr. Rehwinkel (putting his arm around Dr. Atkinson's neck.) Is not that immediate contact? (Laughter.)

Dr. Atkinson. That is the contact of your coat-sleeve.

Dr. Rehwinkel. Then the creasote represents the coat-sleeve, but the coat-sleeve is no fitter for contact than the other.

On motion, Dr. Morrison was invited to read a volunteer on "Operative Dentistry." The paper was devoted to the operation of replantation of teeth. The substance of it is as follows:

The writer stated that he had resorted to the operation several times, and proceeded to give a detailed history of several cases. The first was in the mouth of a delicate lady of forty; the teeth were badly decayed posteriorly, involving the pulp, and the roots covered with a brown, thin scale of tartar. The patient not being able to undergo the operation in the mouth, the tooth was extracted and filled, the tartar removed, and the tooth replaced. For three or four days some pain was suffered, but now, after five months, the tooth is the firmest in the arch, and the gum has a more perfect union than it had for years. Other cases were then detailed, in which the same general plan was pursued, the roots being filled with pure gold wire and the crowns with oxychloride and gold. Some of the cases were mentioned as questionable, or partial failures. One was in the mouth of Dr. Ingersoll, of Keokuk, Iowa,—a right lower first molar, which having an enlarged root, broke in extraction, the posterior root separating, and one-eighth of an inch breaking off from the other root. The molar, or what remained of it, was converted into a bicuspid, filled and replaced, and is doing good service, except on the hardest kind of food.

The writer stated that he had replanted nineteen different teeth, of all classes, all pronounced worthless if treated in the mouth, and of that number only four had been extracted the second time. He took the ground that in all cases where the pulp is dead or beyond restoration to vitality, and in cases with incurable fistulas, the tooth can be more successfully treated and more of the organ saved, taking all risk of breaking into account, in this manner than in any other ; and that though it might be carried to an extreme, yet a large number of teeth removed by accident or otherwise might be restored to usefulness by this method.

Dr. Morgan said that gentlemen seem to insist that death is the result of using oxychloride ; he knows that this is not so. He is careful not to re-expose the pulp, but when he has done so he has found it living twelve months afterwards. Those specimens are pretty, but he fears that they are not practicable. Is not satisfied to fill roots with a plastic material ; does not know what he is doing ; you may pump a portion of the debris backward and you will drive the material out into the soft parts. In the use of oxychloride, there has been more than one case where it has been driven through and necessitated extraction. Any plastic material is liable to this.

Dr. Abbott. Pulps *are* saved with oxychloride in contact with the pulp,—that is, in contact with it except so far as the albumen may seperate them. He had watched and recorded results, and out of seventeen had saved sixteen every time. In one case he had capped three teeth in this way, and filled with amalgam and afterwards with gold, and in doing so the cap flew off, and the pulp was alive and in perfect condition. It makes a great deal of difference how the pulps are handled. They must not be in an irritable condition; and if they are, they must be treated till they can be handled as well as any other wound. Gutta-percha and chloroform will not preserve teeth with him, nor will cork or paper; none of these are half as good as oxychloride.

Dr.——. The coating of albumen is a capping of itself; it prevents the infiltration of fluids; it is just the thing.

Dr. Rawls, of Lexington, spoke of the use of lead wire for filling roots; formed as nearly to the shape as possible by first getting the size of the canal with a piece of cotton wrapped around a broach. It exerts a therapeutic influence in chronic inflammation. Oxychloride in contact with the pulp is not the thing in all cases. The action of the escharotic is to contract the pulp and push itself through. Is the carbolate of albumen soluble or not? If it is, it is either taken up or left *insitu;* and if the former, there must be a space left, which will result in strangulation against the ragged edges of the cavity. We need something that is not an escharotic, that is a good non-conductor, and that will resist the introduction of the permanent filling. This we have not got. Plaster of Paris would be good if it was durable enough. We must protect the pulp from the action of chloride of zinc. Sometimes there is a superabundance of life in the patient that can stand anything; but we do not often have such patients.

The hour fixed for the election of officers having arrived, the association proceeded to the election, with the following result:

*President.*—A. L. Northrop, New York.

*First Vice-President.*—H. J. McKellops, St. Louis.

*Second Vice-President,*—H. A. Smith, Cincinnati.

*Corresponding Secretary.*—J. H. McQuillen, Philadelphia.

*Recording Secretary,*—C. Stoddard Smith, Springfield, Ill.

*Treasurer.*—W. H. Goddard, Louisville.

*Executive Committee.*—New members: L. D. Shepard, Boston; T. L. Buckinham, Philadelphia; and M. H. Webb, Lancaster, Pa.

The action taken at the meeting held in 1872, at which it was voted to hold the meeting of the association in 1876 in Philapelphia, was taken up and ratified.

The meeting then adjourned.

*Evening Session.*

The meeting was called to order at the usual time. The President, Dr. Dean, having left for Europe, the chair was occupied by the vice-president, Dr. Keely.

The Subject of "Operative Dentistry" was still under discussion, and Dr. Rawls continued his remarks. He said that he considered Dr. Atkinson's method of applying oxychloride nearly correct, as he understood it. Dr. Rehwinkel's remarks were also in place as regards soft teeth. Few teeth will bear oxychloride in contact with the pulp even though carbolic acid is used first. If amalgam is applied over the capping it will almost certainly cause the death of the pulp. Bichloride of mercury is formed under the cap; unless the oxychloride is well dried out. Oxychloride is valuable for roots; Introduces it with a few fibers of cotton, and it goes as near to the apex as any other material. Success in capping depends upon the diagnosis. When the pulp is exposed it is in one of three conditions,—either congested, in dead. We must be able to see exactly the condition it is in. It inflamed, or is our duty to save a portion of the pulp alive if possible; it is often wholly destroyed when it should not be. Would cut down to healthy tissue and leave it, and there is thus better chance for success.

Dr. C. R. Butler said that if we must practice by machinery, we might as well be machinists and have done with it. Patients don't like to have their mouths madea machine-shop of. The idea that there must be a machine for everything is unscientific. The ability to cut induces us to cut to too great an extent; this is the case especially with young operators,—they are in great danger of doing damage with the engine. The packing, too, must now be done with machinery. There have been more poor fillings put in during the last ten years by over-packing than by under-packing. Fillings are packed to death. (Applause.) The walls are powdered. They go away from the fillings, and they ought to, for there is no wall there. There is no chemistry about it,—it is miserable mechanics. Hard gold requires great force, and there is no tooth that will stand the continued concussion without becoming powdered. Machinery is doing damage even in the hands of the best men. They don't appreciate the amount of force they are applying. Ho does not argue in favor of any kind of operating, but we may be overdoing the matter, and are more likely to be with machinery. There is no advantage in mixing gold and tin; use either one or the other. The two metals will be likely to give rise to galvanic action. They say that in soft teeth it is more congenial but if cavities are cut out well and well filled, no chemistry will hurt them. Many operators exhaust themselves by having so many appliances and making so many extra movements before they are ready to begin. He then spoke of the use of screws in cuspid teeth, illustrating his remarks by the aid of the black board.

Dr. Abbott. Amalgam fillings sometimes do good service, and many men would save some teeth better with that than with gold. Thousands had better use amalgam, or rather, gutta-percha. But he does not advocate the use of amalgam, and rarely uses it. But sometimes teeth can be saved better with it. Hammering gold against the walls is what produces these chemical changes; the walls are broken, perhaps imperceptible, but the check grows larger. Is not a believer in screws; there is not a case in a thousand where a better filling cannot be made without them. The gold is apt to be loosely packed around them, and is liable if packed hard, to knock the screws loose. Had never had any satisfactory results from their use, and had abandoned them long ago.

Dr. Flagg. It seemed as if, judging from the remarks he had listened to, he was back where he was twenty years ago. If this profession has not changed in that time, it shows that it rests upon a *firm basis*! It requires special skill to work oxychloride of zinc successfully; Dr. Rehwinkel can't work it because he has not the skill to do it, but he has skill to work gutta-percha, and that takes skill. It seemed that the day of "iodine and creasote," of thymol, and those ancient things had passed away. Now the only thing was salicylic acid. That is omega number three: and who could have an omega number three but Dr. Atkinson? He (the speaker) had tried to save more teeth, he supposed, than any other man; he had tried everything, and had saved an everlasting lot of them, and an everlasting lot of them died. He knew they were dead because he

*smelt 'em.* If he thinks that a tooth should be filled with amalgam, he says so. Some plugs *will* fail, though put in as an Abbott or a Butler can put them in; that is, those upon approximal faces. Saving pulps depends upon the patient and upon the operator and largely upon the climatic location. He cannot say with Dr. Abbott that in his region, the miasmatic land between the rivers, he can save sixteen out of seventeen. He is afraid that he loses sixteen out of seventeen. He had formerly used equal parts of creasote and oil of cloves, but now bathes the bottom of the cavity with oil of cloves; it is not escharotic, and it has a sort of satisfying odor, and, by the way, it is very good for wasp-stings. Creasote almost always causes trouble in an irritable tooth; but there is no agent so soothing as oil of cloves. There is nothing better for the aching that sometimes occurs under the application of the dam. This was *his* omega number three.

The speaker made some protracted remarks on the subject of amalgams, though he did not know why they wanted to know about it if none of them used any of it. He is not one of the men that advocate doing good work without regard to price, and then charge seventy-five or one hundred dollars for a filling. A dental depot man had told him that he had sold one thousand ounces of amalgam to one man at one time, and it was not all used by the *poor* dentists either,—those who are not here. Amalgam is the best plastic filling known. The papers read at New York, about which so much had been said, were all bosh, bosh, though we were led to believe that the meeting was the culmination of all dental science. If he had not had special facilities for investigating this subject he would have taken them all for corn. The analyses were far from correct. When men say that fillings won't leak they say what they don't know. He has packed one thousand tubes, and in one tube the filling will leak and in another it will not. The packing is an important matter; if the tube is filled dry there will be no serious leakage, but they all leak some. But amalgam ought not to be washed; if it was the tubes would leak in less than two minutes. He had tried washing in alcohol and also in water, and the difference was just nothing at all, though he had dried his hands and the mass with warm napkins. He would earnestly advise not to wash amalgam ; it must be worked dry. If everything else is equal, the cavity that is most dry will succeed best. The alloys in market are all much alike, and it is not strange that men should be pleased with each. Pressure will not suffice to make a solid filling ; it should be tapped lightly with burnishers. Don't lay it down on the table, but keep the button in your hand to keep it warm ; don't squeeze the last piece hard, but wait till it solidifies, and wipe off the surplus mercury, if any. As to the matter of the drilling machines. he thought the young men could take care of themselves. It was strange how much solicitude was manifested in regard to the young men. He would say let them go on boring, *boring*, BORING ! (Applause.)

The meeting then adjourned. —*Dental Cosmos.*

(TO BE CONTINUED.)

---

